# Accuracy of Surgeon's Estimate of Gross Total Resection After Endoscopic Endonasal Trans-sphenoidal Surgery for Non–Hormone-Producing Pituitary Macroadenomas: Does It Matter?

**DOI:** 10.1227/neuprac.0000000000000269

**Published:** 2026-07-13

**Authors:** Jesse D. Lawrence, Parsa Nilchian, Jeffrey Shi, Steven Zeldin, Natasha Kharas, Rupen Desai, Theodore H. Schwartz

**Affiliations:** 1 Department of Neurosurgery, Weill Cornell Medicine, New York Presbyterian Hospital, New York, New York, USA;; 2 Weill Cornell Medical School, New York, New York, USA;; 3 Department of Neurosurgery, The Mount Sinai Hospital, New York, New York, USA

**Keywords:** Pituitary adenoma, Gross total resection, Endoscopic endonasal surgery

## Abstract

**BACKGROUND AND OBJECTIVES:**

While a surgeon's intraoperative assessment of gross total resection (GTR) has been used to predict recurrence in benign tumors such as meningiomas, its accuracy in pituitary adenoma surgery remains unclear. This study compares intraoperative assessment with postoperative MRI in predicting extent of resection (EOR) and recurrence in patients undergoing endoscopic endonasal resection of nonfunctioning pituitary macroadenomas.

**METHODS:**

A retrospective review was performed on 327 consecutive patients with non–hormone-producing pituitary adenomas resected by a single surgeon. At the conclusion of each operation, the surgeon recorded the intraoperative assessment of EOR. All patients underwent pre- and postoperative MRI, and EOR was independently assessed by neuroradiology. Radiographic recurrence and additional treatment need were correlated with intraoperative assessment and postoperative MRI findings.

**RESULTS:**

Intraoperatively, the surgeon overestimated GTR rates. While intraoperative assessment GTR was reported in 90%, MRI revealed GTR in 72%. The positive predictive value of intraoperative assessment was 74% and the negative predictive value was 84%. After 40.7 months of mean follow-up, there was a 16.7% recurrence rate. Patients with intraoperative assessment GTR and subtotal resection (STR) recurred at rates of 14% and 39%, respectively. Patients with radiographic GTR and STR recurred at rates of 6% and 44%. Discordant cases with intraoperative assessment GTR and MRI STR (n = 57) recurred at 42%, indicating MRI was a more accurate recurrence predictor. Discordant cases with intraoperative assessment STR and MRI GTR (n = 6) recurred at a rate of 0%, indicating the higher accuracy of postoperative MRI. The sensitivity and specificity of intraoperative assessment for predicting recurrence were 24% and 93%, respectively. Both sensitivity and specificity of postoperative MRI for predicting recurrence were 78%.

**CONCLUSION:**

Postoperative MRI was more accurate at establishing GTR, identifying residual tumor, and predicting recurrence compared with the surgeon's intraoperative assessment. This study supports the limited value of intraoperative EOR assessment compared with imaging.

**ABBREVIATIONS:**
EOR: extent of resection
GTR: gross total resection
STR: subtotal resection.

Trans-sphenoidal surgery remains the primary treatment modality for most non–hormone-producing macroadenomas. Although most adenomas are benign, slow growing tumors, it is well-established that extent of resection (EOR) is an independent predictor of recurrence. Thus, the goal of surgery is maximal safe cytoreduction.^1-5^ The gold standard for measuring EOR remains postoperative MRI, which is highly accurate even if performed immediately after surgery.^6,7^ Although intraoperative MRI is available at some institutions, the decision to pursue more aggressive surgery is often based on the surgeon's estimate of EOR, partly because the advent of angled endoscopes has greatly facilitated cavity inspection.^8^ Moreover, it is theoretically possible that the surgeon could see small remnants of tissue that are below the resolution of an MRI scan, which might make the surgeon's estimate more accurate in certain circumstances.

Surgeons' estimate of EOR has been shown to be notoriously unreliable for several brain tumors, including gliomas and meningiomas.^9,10^ Most studies show a discordance rate of roughly 20% with most surgeons overestimating the rate of gross total resection (GTR).^9,10^ However, whether the same holds true for pituitary macroadenomas in the endoscopic era has not been studied. Thus, the first goal of this article was to determine the accuracy of the surgeon's intraoperative assessment of GTR by calculating the positive and negative predictive values compared with early postoperative imaging. However, the second important question is whether such a surgical underestimation, if it exists, is clinically relevant. To answer this second question, we examined the long-term recurrence rate to determine whether patients who were presumed to have a GTR by the surgeon's estimation but had a subtotal resection (STR) by postoperative MRI had a higher recurrence rate. Similarly, given the possibility that the surgeon might appreciate some residual tumor that is missed by postoperative MRI, we also examined the opposing possibility in which the surgeon estimated STR, but the postoperative MRI revealed a GTR and compared the recurrence rates of these 2 subgroups.

## METHODS

This study was approved by our Institutional Review Board. We performed a retrospective review of prospectively acquired database of all 381 consecutive non–hormone-producing pituitary macroadenoma patients operated on by the senior author between January 2013 and September 2023. There were no exclusions based on tumor size, invasion, or patient demographics. Patients were excluded only if clinical records, intraoperative assessment of EOR, postoperative imaging, or follow-up data were incomplete. At the conclusion of each operation, the operating surgeon noted whether a GTR or subtotal STR had been achieved. Patient consent was obtained for all procedures reported in this study.

Intraoperative assessment of EOR was performed using endoscopic visualization of the surgical cavity at the conclusion of tumor removal. Angled endoscopes were used when necessary to enhance the visualization of regions not adequately visualized with a straight endoscope, including the diaphragma sellae and the walls of the cavernous sinus. The surgeon's intraoperative assessment of EOR was based on inspection of these structures for visible residual tumor. The EOR was then determined by a neuroradiologist who read the postoperative MRI, in comparison with the preoperative MRI. Imaging consisted of T1-weighted sequences acquired with and without contrast. Postoperative MRI was obtained within 48 hours of surgery, a time window commonly used in pituitary adenoma surgery to assess EOR. When compared with preoperative imaging, early postoperative MRI has been shown to provide a reliable assessment of residual tumor after trans-sphenoidal resection.^7^ The neuroradiologists were not aware of the surgeon's intraoperative estimation of the EOR. Imaging results were dichotomized into GTR or STR. Patients were then followed to determine whether there were any tumor recurrences or delayed postoperative treatment such as reoperation or radiation therapy to treat progressive growth. Tumor outcome was defined as either recurrence or progression. Recurrence was defined as the appearance of new enhancing tumor (>0.1 cm^3^) following GTR, whereas progression (or regrowth) was defined as a ≥25% increase in residual tumor volume following STR. Throughout the Results, the term “recurrence” refers to tumor reappearance following GTR, whereas “progression” refers to interval growth of known residual tumor following STR; when reported together, these outcomes are referred to as “tumor recurrence or progression.”

## RESULTS

There were 327 patients who met inclusion criteria for this study. Baseline demographic and radiographic characteristics are summarized in Table 1; the cohort was 53% male with a mean age of 58 ± 14 years and mean tumor volume of 5.6 ± 6.1 cm^3^, and 13% underwent reoperation. Tumors most commonly involved the sellar/suprasellar region (62%), with cavernous sinus involvement in 33% (right, 19%; left, 14%) and extrasellar involvement in 5%. Of these 327 patients, 294 patients were classified by the surgeon by intraoperative assessment as GTR (90%). Neuroradiology evaluation of the postoperative MRI revealed GTR in 220 patients (72%). If we use the postoperative MRI as the gold standard, the positive predictive value of the surgeon's intraoperative assessment of GTR was 74% and the negative predictive value was 84%.

**TABLE 1.** Baseline Demographic and Radiographic Characteristics of the Study Cohort

No. of patients	327
%Male	53
Age ± SD (y)	58 ± 14
Tumor volume (cm^3^)	5.6 ± 6.1
Reoperation (%)	13

**FIGURE 1.**
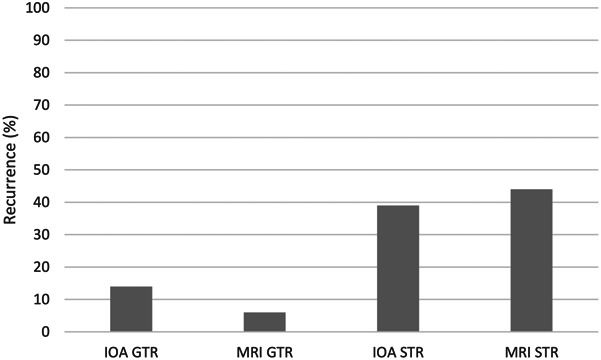
Comparative recurrence rates of intraoperative assessment compared with post-operative MRI. GTR, gross total resection; IOA, intraoperative assessment; STR, subtotal resection.

**FIGURE 2.**
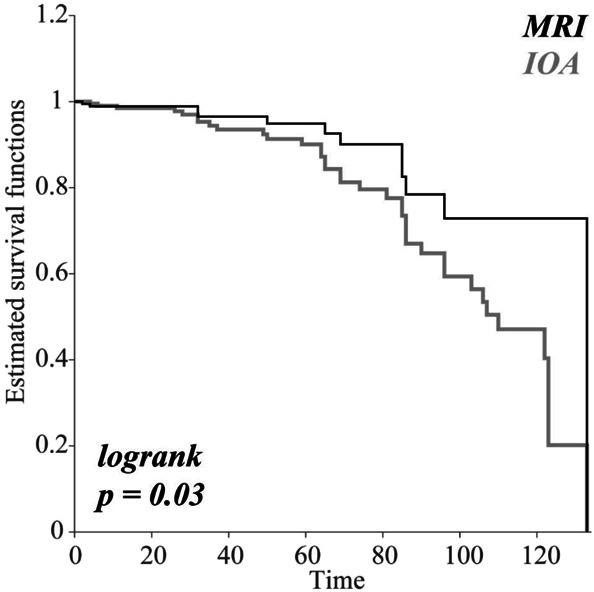
Progression-free survival Kaplan–Meier plots on intraoperative assessment gross total resection with post-operative MRI assessment of extent of resection. IOA, intraoperative assessment.

**FIGURE 3.**
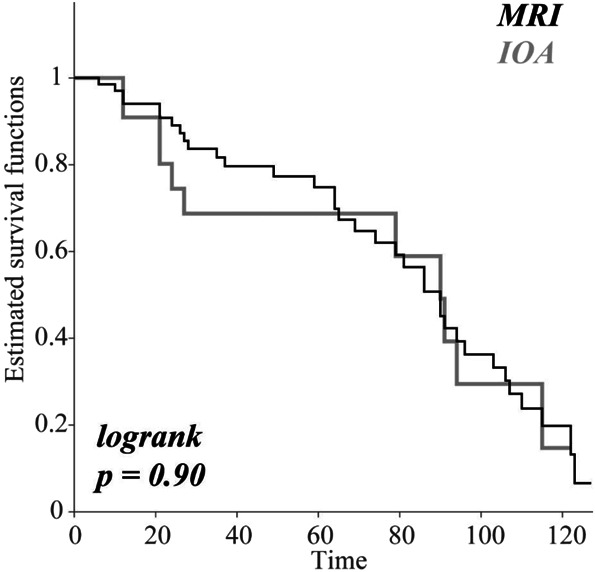
Progression-free survival Kaplan–Meier plots on intraoperative assessment subtotal resection with post-operative MRI assessment of extent of resection. IOA, intraoperative assessment.

The overall tumor recurrence or progression rate was 16.7% with an average follow-up of 40.7 months. Patients with intraoperative assessment of GTR and STR had tumor recurrence and progression rates of 14% and 39%, respectively. Patients with MRI determined GTR and STR had recurrence or progression rates of 6% and 44%, respectively (Figure 1). Progression-free survival plots reveal that MRI-determined GTR is more accurate than intraoperative assessment at predicting recurrence (hazard ratio = 1.95, 95% CI: 1.07-3.57; *P* = .03; Figure 2). On the other hand, intraoperative STR and MRI-determined STR were equally good predictors of progression (Figure 3). The sensitivity and specificity of intraoperative assessment to predict recurrence and progression were 24% and 93%, respectively. Both the sensitivity and specificity of postoperative MRI to predict recurrence and progression were 78%.

**FIGURE 4.**
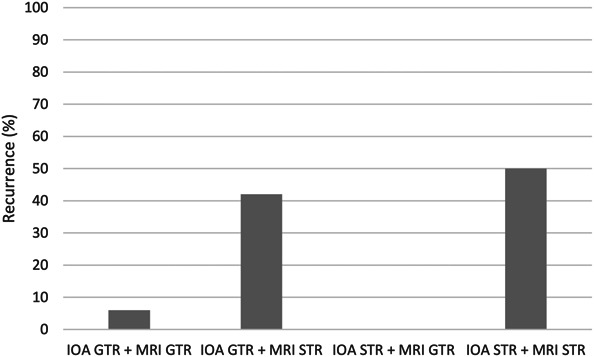
Comparative recurrence rates of concordant and discordant extent of resection determinations. GTR, gross total resection; IOA, intraoperative assessment; STR, subtotal resection.

There were 57 discordant cases with intraoperative assessment of GTR but postoperative MRI showing STR. These patients recurred at a rate of 42% (Figure 4). On postoperative MRI review, residual tumor was most commonly located within the cavernous sinus (40%), followed by the sella (30%), suprasellar region (21%), and other extrasellar locations (9%) (Table 2). There were 6 discordant cases with intraoperative assessment of STR and postoperative MRI showing GTR. There were no progressions in this group (Figure 4).

**TABLE 2.** Location of Residual Tumors in Discordant Cases Classified as Intraoperative Gross Total Resection But as Postoperative Subtotal Resection

Total no. of cases	57
Left cavernous sinus	12 (21%)
Right cavernous sinus	11 (19%)
Sellar	17 (30%)
Suprasellar	12 (21%)
Other extrasellar	5 (9%)

## DISCUSSION

### Key Results

In this cohort of 327 patients, surgeons overestimated GTR compared with postoperative MRI (90% vs 72%), with intraoperative assessment showing limited predictive accuracy. Postoperative MRI more reliably stratified recurrence/progression risk, particularly in discordant cases, where patients deemed GTR intraoperatively, but STR on MRI had high rates of recurrence/progression, while no events occurred when MRI confirmed GTR despite intraoperative STR.

### Interpretation

Several studies have shown the importance of EOR in preventing recurrence in patients with non–hormone-producing pituitary macroadenomas. Previous studies have demonstrated that patients with STR progress at rates of 24% to 58% whereas those with GTR recur at 4.7% to 20% when postoperative MRI is used as a metric of EOR.^1-5,11,12^ Moreover, the volume of residual tumor is also important with larger volume remnants leading to higher rates of progression.^3,4^ Thus, at the time of surgery, surgeons often have to make intraoperative determinations of EOR, now mostly performed with an endoscope, since safely maximizing GTR is the goal of surgery. For this reason, intraoperative MRI has been applied to achieve this goal.^8,13,14^

We demonstrate that the surgeon's intraoperative assessment of EOR, even with the use of an endoscope, is an unreliable predictor of residual tumor found on postoperative MRI. In cases discordant for EOR, residual tumor on postoperative MRI was most commonly located within the cavernous sinus, followed by intrasellar and suprasellar regions. This pattern suggests that parasellar tumor extension into anatomically constrained regions may limit intraoperative visualization and contribute to overestimation of GTR during surgery. Moreover, postoperative MRI assessment of EOR is a more accurate predictor of tumor recurrence/progression compared with the surgeon's intraoperative estimation. Although intraoperative judgement will always be the sine qua non of surgical decision making, as with other tumor pathologies, it falls short of accurately determining EOR and long-term recurrence/progression rates. The low sensitivity of intraoperative assessment for predicting tumor progression likely reflects a combination of overestimation of GTR and the inherent limitations of endoscopic visualization. Residual tumor may remain in anatomically concealed or surgically constrained regions such as the cavernous sinus, suprasellar cistern, or along the diaphragma sellae, where complete resection may be unsafe or visually obscured. As a result, small residual deposits not appreciated intraoperatively may be detected on postoperative MRI and subsequently demonstrate progression over time.

Intraoperative MRI is one of the most widely studied adjunct capable of improving both the intraoperative assessment of EOR and the achievement of GTR in pituitary adenoma surgery.^15-17^ By allowing real-time identification of residual tumor, intraoperative MRI has been shown to increase rates of additional resection in select cases.^15^ However, its clinical adoption remains limited because of substantial cost, infrastructure demands, and prolonged operative time, and it is not routinely available at many centers, including our own. As a result, intraoperative decision making at most institutions continues to rely primarily on endoscopic visualization, underscoring the importance of accurate postoperative MRI for definitive assessment of EOR and long-term prognostication.

The relevance of surgeons' estimation of EOR has been investigated in other tumor pathologies, most notably in meningiomas. The earliest and most highly used grading scale for meningiomas has been the Simpson grading scale. Investigations into this grading scale show that it correlates moderately well with recurrence, but not as well as postoperative MRI assessment of EOR.^12,18-21^ Furthermore, in 8% of cases, surgeons were inaccurate in identifying Simpson grade when comparing with postoperative MRI results.^19^ Thus, in other benign tumors, postoperative MRI has been shown to be a better predictor of tumor recurrences compared with surgeons estimation.^21^

The intraoperative assessment of resection was also investigated in awake glioma surgery. Analyzing both continuous EOR and dichotomous (GTR vs STR) categories of resection, Lau et al^22^ found inaccurate assessment of resection in nearly 1 of every 5 cases. Furthermore, they showed that the accuracy of assessment improves with surgeon experience, which may take 10 years to develop. Intraoperative assessment of EOR, like any other surgical observation or skill, improves over the course of a surgeon's career, which often spans decades.^23^ Nevertheless, as surgeons cannot see through opaque tissues, postoperative MRI will always provide a better metric of EOR.

### Generalizability

Although this is a single-center, single-surgeon study, the large consecutive cohort and consistent surgical and imaging protocols reduce technical variability, supporting the internal validity of the findings; however, generalizability to other practice settings, surgeons with different experience levels, or institutions using alternative imaging strategies may be more limited.

### Limitations

Although this study might be interpreted as support for the routine use of intraoperative MRI, it was not designed to address this question. For example, just because residual tumor is identified on MRI does not mean it can be safely removed. It may be that intraoperative endoscopic inspection is adequate to identify the presence of most instances of residual tumor that can be safely removed but falls short of identifying all areas of residual tumor. In addition, formal preoperative grading of cavernous sinus invasion using the modified Knosp classification was not systematically available in this retrospective cohort. Incorporation of standardized Knosp grading in future studies would provide a more granular assessment of tumor invasiveness and further contextualize the relationship between intraoperative assessment, EOR, and postoperative imaging findings. Further studies will have to be performed that directly address this question.

While postoperative MRI scans are currently the gold standard for visualizing residual tumor and predicting recurrence/progression, surgeons will increasingly rely on molecular forms of imaging, rather than gross assessments. These will include targeted radiolabeled or fluorescent probes that can be seen directly during surgery, such as 5-aminolevulinic acid for high-grade gliomas, or with sensitive imaging techniques after surgery, such as [Cu64]DOTATATE imaging for meningiomas.^24,25^ Such tests are more sensitive at identifying residual tumor and will flatten the learning curve rendering surgeon's intraoperative white light estimations less critical. These, in combination with molecular predictors such as Ki-67, p53, cadherin, pituitary tumor–transforming gene, matrix metalloproteinase-9, and epidermal growth factor receptor, among others will eventually become the gold standard.^26^

## CONCLUSION

Surgeon estimation of the EOR by an experienced pituitary surgeon fails to accurately determine tumor resection in nearly 25% of cases when compared with postoperative MRI. Intraoperative assessment is also inferior compared with postoperative MRI at predicting recurrences.
